# Clinical manifestations and treatment management of hospitalized patients with zinc phosphide poisoning, Mazandaran Province, Northern Iran

**DOI:** 10.1186/s12873-022-00661-1

**Published:** 2022-06-11

**Authors:** Seyed Khosro Ghasempouri, Zakaria Zakariaei, Seyed Mohammad Hoseininejad, Fatemeh Chinian, Mostafa Soleymanii, Seyedeh Masoumeh Pashaei, Mahdieh Sadeghi

**Affiliations:** 1 Department of Forensic Medicine and Toxicology, School of Medicine, Mazandaran University of Medical Sciences, Sari, Iran; 2 Toxicology and Forensic Medicine Division, Orthopedic Research Center Communicable Diseases Institute, Imam Khomeini Hospital, Mazandaran University of Medical Sciences, P. O box: 48166-33131Sari, Mazandaran, Iran; 3 Gut and Liver Research Center, Mazandaran University of Medical Sciences, Sari, Iran; 4 Student Research Committee, Mazandaran University of Medical Sciences, Sari, Iran; 5 Iranian National Registry Center for Lophomoniasis and Toxoplasmosis, Imam Khomeini Hospital, Mazandaran University of Medical Sciences, Sari, Iran; 6 Department of Emergency Medicine, Mazandaran University of Medical Sciences, Sari, Iran

**Keywords:** Zinc phosphide, Poisoning, Suicide, Clinical manifestations

## Abstract

**Background:**

Zinc phosphide (ZnP) is a dark gray crystalline compound used as a rodenticide against rodents such as mice. ZnP poisoning may be accidental or suicidal. The aim of this study was to investigate the clinical manifestations and treatment management of hospitalized patients with ZnP poisoning in Mazandaran Province, northern Iran.

**Methods:**

Between 2013 and 2017, a cross-sectional study was performed on hospitalized patients with ZnP poisoning who were referred to two training hospitals in Mazandaran Province, northern Iran.

**Results:**

A total of 127 patients participated in this trial, including 71 (55.9%) men and 56 (44.1%) women. The patients’ average (standard deviation) age was 25.5 (±16.82) years, and it took 2.18 (±2.23) hours to refer them to the hospital. There were 42 (33%) cases with less than one package, 9 (7%) cases with several packages, and 76 (60%) cases with no particular usage.

**Conclusions:**

This study has shown that ZnP poisoning may be asymptomatic initially or with mild clinical symptoms that may gradually worsen. Therefore, hospitalization and obtaining a history and a careful physical examination should be considered.

## Introduction

Zinc phosphide (ZnP) and other compounds containing phosphine gas are effective fumigants and rodenticides that are widely utilized in many countries, particularly in developing countries. After consumption, phosphides are converted by gastric acid into phosphine gas, the main toxic substance. Phosphine gas then enters the bloodstream from the gastrointestinal tract. In humans, phosphine is a highly toxic gas that affects the body through a number of mechanisms, including inhibition of cytochrome c oxidase, oxidative respiration, the formation of free radicals via increased lipid peroxidation, and acetyl cholinesterase inhibition (AChE) [1, 2]. Phosphine gas released from refined grains in silos and other agricultural areas can be toxic by inhalation, and poisoning by it happens quickly (usually within 30 minutes of exposure) when it comes into contact with the skin [3, 4].

Phosphine gas generally affects organs such as the gastrointestinal (GI), cardiovascular, respiratory, hepatobiliary, and hematologic systems, as well as causes electrolyte and metabolic disorders [1, 2]. Some of the serious symptoms of phosphine gas poisoning are hypotension, pulmonary edema, congestive heart failure, cardiac arrhythmia, cardiovascular collapse, and acute kidney injury [1, 5]. Hepatotoxicity and intravascular hemolysis with methemoglobinemia, as well as renal failure, are less common findings [6].

Since no specific antidote has been discovered, supportive care is the mainstay of management. Despite some efforts to develop more effective therapies and drugs for treatment, the fatality rate remains high, particularly for aluminum phosphide (AlP) toxicity. Although AlP and ZnP both produce phosphine gas in the human body, some symptoms and the mortality rate are different between these two metal phosphides [7, 8].

ZnP is a black powder or gray crystalline compound [1, 2]. Due to its low cost and widespread availability, ZnP is commonly used as a household rodenticide in developing countries due to its low cost. There are limited reports and clinical studies on ZnP poisoning in humans [5, 9–13]. The diagnosis of phosphide poisoning is made by history and clinical signs. Exposed patients may also show hypokalemia and elevated lactate levels, although these findings are not diagnostic. According to case reports, phosphide is radiopaque in x-ray imaging, so abdominal radiographs can help confirm the diagnosis [14]. Consequently, the current study was performed to investigate the clinical findings and early management of patients with ZnP poisoning who were referred to two training hospitals in Mazandaran Province, northern Iran.

## Patients and methods

This cross-sectional study was approved by the Mazandaran University of Medical Science Ethics Committee (IR.MAZUMS.REC.1398.1717) and was carried out in accordance with the Helsinki Declaration Principles.

The current research is a cross-sectional study that was performed on patients with ZnP poisoning who were referred to two training hospitals (Imam Khomeini and Razi) in Mazandaran Province, northern Iran, from 2013 to 2017. Inclusion criteria included all patients with a history of ZnP ingesting, laboratory tests such as arterial blood gases and abdominal and pelvic x-rays. Exclusion criteria included incomplete information registration, dissatisfied patients, and those who did not cooperate.

### Statistical analysis

The study’s statistical population included 127 ZnP poisoning patients, and data collection procedures included a checklist that contained personal and social information such as age, gender, and marital status. The amount of ZnP consumed, the time to refer to the emergency room, vital signs and clinical manifestations such as nausea, vomiting, abdominal pain, dyspnea, drowsiness, respiratory distress, palpitations, and level of consciousness were all recorded, as were diagnostic and therapeutic processes such as pulse oximetry, serum electrolyte levels, blood biochemistry, and the need for intubation.

The software used in this study was SPSS v.20, and a *P* value < 0.05 was considered a significant level. Frequency was used to show qualitative variables, and mean and standard deviation were used to express quantitative variables. A Pearson or Spearman coefficient was calculated to examine the correlation between quantitative variables, and a Chi-Square test was used to correlate the qualitative variables.

## Results

This cross-sectional study was performed to investigate the clinical manifestations of patients with ZnP poisoning, and 127 patients were studied, in which only one death was seen among the patients. Among the patients, 71 (55.9%) were male and 56 (44.1%) were female. The mean (standard deviation) age of patients was 25.5 (±16.82) years, with a minimum age of 2 years and a maximum age of 81 years. Also, 48 cases (37.8%) were single and 79 cases (62.2%) were married (Tables 1, 2 and 3).

**Table 1:** Demographic index of evaluated patients

Variables		Frequency (%)
Gender	Men	71 (55.9)
	Women	56 (44.1)
Marital status	Single	48 (37.8)
	Married	79 (62.2)

**Table 2:** Mean and standard deviation of patients’ vital signs

Variables	Lowest	Highest	Mean (SD)
Temperature	37.00	38.20	36.7082 (1.43867)
Systolic blood pressure	75.00	160.00	116.012 (19.40022)
Diastolic blood pressure	50.00	90.00	72.2000 (11.96543)
Number of breaths	12.00	33.00	18.9300 (3.7245)
Heart rate	65.00	175.00	88.7400 (18.35112)
Spo2	88.00	100.00	98.1000 (2.75462)

**Table 3:** The frequency of the signs and symptoms during hospitalization in evaluated patients

Variables		Frequency (%)
Signs and symptoms	Nausea &Vomiting	61 (48)
	Abdominal Pain	33 (25.9)
	Respiratory Disorder	3 (2.36)
	Drowsiness	5 (3.9)
	Palpitation	1 (0.787)
	Abdominal pain,Nausea and Vomiting	18 (14.1)

The average (standard deviation) time of referring to the hospitals was 2.18 (±2.23) hours, with a minimum of half an hour and a maximum of 10 hours. In 42 cases (33%), the consumption amount was less than one package (each package of ZnP is 10 g) and more than one package in nine cases (7%). In addition, in 76 cases, it was unclear (60%).

Vital signs and serum electrolyte levels were evaluated, the results of which are shown (Tables 3, 4). Also, 5 cases (3.9%) of patients had dizziness, 1 case (0.787%) had diarrhea, 1 case (0.787%) had weakness and lethargy, 1 case (0.787%) developed a seizure, 4 cases (3.15%) had headaches, and 2 cases (1.57%) of patients were pregnant. In terms of symptoms and laboratory findings, one case (0.787%) had a temperature of more than 37.8 °C, two cases (1.57%) had blood pressure below 90 mmHg, and eight cases (6.29%) had blood pressure (BP) above 140 mmHg. More than 20 breaths/minute were recorded in 15 patients (11.8%), and a heart rate (HR) of more than 100 beats per/minute was reported in 15 cases (11.8%). Hypernatremia was observed in 7 (5.5%) of the patients, whereas hyperkalemia was found in 1 (0.787%).

**Table 4:** Pearson correlation was performed between different variables in patients, the results of which are shown in the table below

		Age	Referred time	Usage rate	Temperature	Blood pressure	Respiratory rate	Heart rate	Hypernatremia	Hyperkalemia	Acidosis
Age	Correlation Coefficient	1.000									
	Sig. (2-tailed)	.									
Referred time	Correlation Coefficient	.001	1.000								
	Sig. (2-tailed)	.996	.								
Usage rate	Correlation Coefficient	−.050	.180	1.000							
	Sig. (2-tailed)	.730	.211	.							
Temperature	Correlation Coefficient	−.154	−.097	−.175	1.000						
	Sig. (2-tailed)	.287	.502	.224	.						
Blood pressure	Correlation Coefficient	.270	−.162	.020	−.025	1.000					
	Sig. (2-tailed)	.057	.262	.891	.865	.					
Respiratory rate	Correlation Coefficient	−.378	−.207	−.066	−.053	−.223	1.000				
	Sig. (2-tailed)	.007	.149	.651	.716	.120	.				
Heart rate	Correlation Coefficient	−.346	−.134	−.270	−.053	−.223	.621				
	Sig. (2-tailed)	.014	.352	.058	.716	.120	.000				
Hypernatremia	Correlation Coefficient	.127	.032	.110	.117	.127	−.201	−.201	1.000		
	Sig. (2-tailed)	.378	.824	.448	.420	.380	.162	.162	.		
Hyperkalemia	Correlation Coefficient	.035	.174	.124	−.020	−.025	−.053	−.053	.117	1.000	
	Sig. (2-tailed)	.811	.227	.390	.888	.865	.716	.716	.420	.	
Acidosis	Correlation Coefficient	−.070	−.142	.040	.062	.213	−.168	−.003	.316	.062	1.000
	Sig. (2-tailed)	.627	.326	.782	.668	.137	.245	.716	.982	.668	.

## Discussions

In our study, with the exception of one case that died due to multiple organ failure, most patients had gastrointestinal symptoms, and no hepatic or renal failure was reported. The clinical manifestations in patients were as follows: nausea and vomiting in 61 cases (48%), abdominal pain in 33 cases (25.9%), nausea and vomiting with abdominal pain in 18 cases (14.1%), five patients had drowsiness, and three patients developed respiratory disorders. Hydration, insertion of a nasogastric tube, administration of charcoal-sorbitol, pantoprazole ampoule, and correction of acidosis with sodium bicarbonate were among the treatments provided to the patients.

The mortality rate in our study was 0.787% (1 patient), and this patient was an 81-year-old man (the oldest case in the study) who had an underlying disease. The cause of low mortality can be due to the humidity of the air in the study area, the low quality of the powder or its improper packaging, as well as the following early access to hospital care and the possibility of consuming small amounts of ZnP powder.

In 2017, Trakulsrichai et al. identified 455 poisoned people, 60.5% of whom were male, with a mean age of 39.91 years. The most common method of consumption was oral (99.3%). Most patients initially had normal vital signs and oxygen saturation with no altered mental status. Three of the most common clinical symptoms were gastrointestinal (68.8%), cardiovascular (22%) and pulmonary (13.8%). Most patients initially had normal blood test results and normal chest radiographic findings. The average hospitalization was 2 days, and the mortality rate was 7%. Approximately 70% of patients underwent gastric lavage and single-dose activated charcoal. A total of 31 cases underwent intubation and mechanical ventilation. Inotropic drugs were prescribed in 4.2% of cases [7].

Also, Nekoukar et al. reported a young man who, after intentional consumption of an unknown amount of ZnP powder, developed symptoms of nausea, vomiting, palpitations, jaundice, and liver and kidney failure, and died a few days later. They have concluded that in patients with a history of ingestion of rodenticide compounds, particularly ZnP, an arterial blood gas (ABG) and an abdominal x-ray are required, and GI decontamination with polyethylene glycol (PEG) should be considered in the presence of radiopaque substances [14, 15].

In 1998, Chugh et al. studied 20 cases in a study called “Symptoms of ZnP poisoning.” The most common manifestations were vomiting, abdominal pain, palpitations, sweating, dyspnea, tachypnea, metabolic acidosis, shock, and hypotension. There have been five deaths [5].

Gunaratne et al. reported severe ZnP poisoning in a 14-year-old girl who attempted suicide with phosphide and was referred for vomiting, then developed metabolic acidosis, acute pulmonary edema, acute renal failure, acute hepatic failure, and coagulopathy. She also had hyperglycemia, which is rare and is a poor prognostic indicator of exposure to phosphine gas. Upon admission to the hospital, the patient was conscious with a Glasgow Coma Scale (GCS) of 15, a HR of 80 beats/minute, and a BP of 70/100 mmHg [16].

In 2018, Hassanzadeh et al. reported a case of acute renal failure (ARF) and cardiac arrest following the consumption of ZnP rodenticide in Yasuj city. The patient was an 18-year-old man who used the rodenticide to commit suicide. They concluded that ZnP intoxication could lead to ARF [17].

## Conclusion

Although many studies have shown that ZnP poisoning can be asymptomatic or cause mild clinical symptoms at first, it is associated with a poor prognosis and a variety of complications, including multi-organ failure and mortality, in some cases. To prevent undesirable consequences, timely and appropriate diagnosis and treatment are recommended in cases of phosphide compound poisoning.

## Data Availability

The datasets used and analyzed during the current study available from the corresponding author on reasonable request.

## Abbreviations

ZnP: Zinc phosphide
AChE: Acetyl cholinesterase inhibition
GI: Gastrointestinal
AlP: Aluminum phosphide
ABG: Arterial blood gas
PEG: Polyethylene glycol
GCS: Glasgow Coma Scale
HR: Heart rate
BP: Blood pressure
ARF: Acute renal failure
